# Correlation Between Postoperative Vitreous Hemorrhage and Preoperative Evaluation of Optical Coherence Tomography Angiography in Proliferative Diabetic Retinopathy Surgery

**DOI:** 10.1155/joph/7839246

**Published:** 2025-07-05

**Authors:** Yusuke Haruna, Mizuki Tagami, Gen Kinari, Atsushi Sakai, Shigeru Honda

**Affiliations:** Department of Ophthalmology and Visual Sciences, Graduate School of Medicine, Osaka Metropolitan University, 1-5-7 Asahimachi, Abeno-ku, Osaka-shi, Osaka 545-8585, Japan

**Keywords:** diabetic retinopathy, optical coherence tomography angiography, postoperative complication, surgical retina, vitreous hemorrhage

## Abstract

**Purpose:** To measure and compare the extent of retinal neovascularization using optical coherence tomography angiography (OCTA) between patients with good postoperative outcomes for proliferative diabetic retinopathy (PDR) requiring surgical treatment and patients with vitreous hemorrhage (VH).

**Methods:** This retrospective study included patients who were diagnosed with PDR between January 2022 and December 2023 and underwent vitreous surgery. Cases that developed postoperative VH were classified as the VH group, and cases with good postoperative progress were classified as the control group. The extent of retinal neovascularization was measured from preoperative and postoperative images of the two groups taken by OCTA measured with a widefield optical coherence tomography (Canon, Xephilio OCT-A1), and a comparative study was conducted.

**Results:** The VH group consisted of 8 patients with 11 eyes (4 men and 4 women) with a mean age of 49.7 ± 14.2 years, while the control group consisted of 23 patients with 26 eyes (19 men and 4 women) with a mean age of 56.9 ± 11.8 years. The preoperative retinal neovascular area was 50,233.7 ± 38,581.1 (pixels) in the VH group and 17,155.4 ± 27,950.2 (pixels) in the control group, showing a significant difference (*p*=0.046). The postoperative retinal neovascular area was 36,315.7 ± 44,311.8 (pixels) in the VH group and 2261.0 ± 9072.2 (pixels) in the control group, showing a significant difference (*p*=0.046), but there was no significant difference in the reduction rate of the neovascular area before and after surgery (*p*=0.30).

**Conclusions:** In PDR developing VH after vitrectomy surgery, the appearance of neovascularization seen on pre- and postoperative OCTA is significantly more extensive than in cases that do not develop postoperative VH, and OCTA may be useful for preoperative evaluation.

## 1. Introduction

Proliferative diabetic retinopathy (PDR) is one of the major diseases that threaten visual function in the world [1]. The global prevalence of diabetic retinopathy (DR) in diabetic patients was estimated at 22%, with about 6% exhibiting vision threatening [2]. Of course, as previously reported, photocoagulation (PC) of neovascularization (NV) in PDR is the most important basic treatment, but it can be difficult if vitreous hemorrhage (VH) is also present [3]. It is therefore a disease that requires accurate diagnosis and prompt treatment, including surgical intervention including small gaze pars prana vitrectomy, and vision can often be preserved if appropriate intervention is performed [4–10].

Traditional imaging modalities for DR risk stratification have significant limitations. For instance, fundus photography is limited in its ability to discern finer vascular details. Fluorescein angiography (FA) is the most popular method of evaluating retinal blood flow [11]. However, in recent years, highly noninvasive optical coherence tomography angiography (OCTA) has been put to practical use in clinical practice [12, 13]. In particular, although there has been an increase in reports of enface images taken with wide-angle OCTA, there are few studies that focus on NV in the vitreous cavity [12, 14–16].

In addition, Khalid et al. reported that OCTA had a higher detection rate for NV than ophthalmoscopy [17].

However, few studies have examined postoperative evaluations of PDR including re-VH using OCTA during the perioperative period of vitreous surgery [18, 19].

In this retrospective case series, we used wide-angle OCTA by using the wide-angle OCTA enface method to quantify enface NV in the vitreous cavity and predict VH after vitreous surgery for PDR.

## 2. Methods

Prior to this study, we obtained approval from the Ethics Committees of the Osaka Metropolitan University (OMU) (approval no. 2023-043), Japan. Informed consent was obtained from all patients prior to inclusion. The study protocol was a single-center study with a retrospective chart review of all patients who met the study criteria described below.

From January 2022 to December 2023 at the Department of Ophthalmology, OMU patients underwent 25 or 27-gauge transconjunctival sutureless pars plana vitrectomy (PPV) with/without phacoemulsification and intraocular lens implantation (PEA + IOL) for PDR. Regarding the indication for vitrectomy in PDR eyes without traction retinal detachment (TRD) or VH, these cases primarily underwent surgery for persistent fibrovascular proliferation with a high risk of progression to TRD or VH, despite prior antivascular endothelial growth factor (VEGF) therapy and panretinal PC. We classified patients who developed VH after surgery as the VH group and those who did not develop VH as the control group. VH was diagnosed by the examining physician using a slit lamp examination or ultrasound examination. We used enface OCTA (details will be explained later) to compare whether there was a difference in NV between the two groups. The VH group was defined as cases in which VH occurred after PPV during the follow-up period. Postoperative OCTA images of the VH group were taken within 3 months after surgery. In most cases, VH occurred 1 month or more after surgery, and OCTA images were taken before the onset of VH. There were two cases in which VH occurred within 1 month after surgery, and both disappeared within about 1 month, so OCTA images were taken after that.

The inclusion criteria were as follows: (1) patients who underwent PPV for PDR and (2) patients who underwent examinations and detected retinal NV by enface OCTA (Xephilio OCT-S1, Canon, Tokyo, Japan) before and after treatment.

The exclusion criteria were as follows: (1) patients who responded to OPTOUT and refused data extraction. (2) Cases in which the investigators determined that there were motions or physical artifacts that made quantitative evaluation of enface OCTA difficult (e.g., eyes with dense VH obscuring the fundus).

Best-corrected visual acuity (BCVA) results were converted to the logarithm of the minimum angle of resolution (log MAR) based on the previous report [20].

### 2.1. Image Analysis

**Figure 1:**
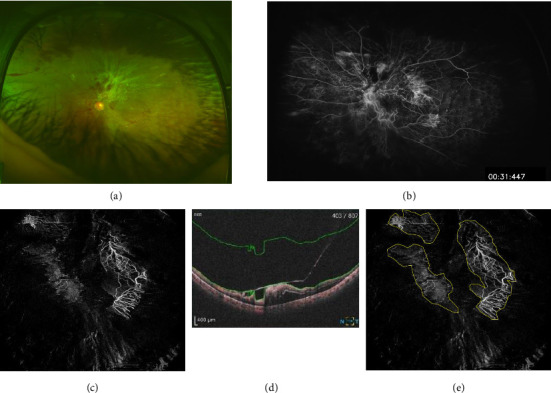
Representative case. (a) Optos image. (b) Fluorescein angiography image using Optomap 200Tx. (c) Enface OCTA images. (d) B-scan image. The area was specified from the surface of the internal limiting membrane (ILM) to 2000 μm above the ILM. (c) is the enface OCTA image. The blood flow signal present within the area selected in the B-scan image (d) was displayed in the enface image. (e) Using Image J, the blood flow signal (NV) was manually surrounded (yellow line), and the total number of pixels within it was calculated. In this case, it was 38,950 pixels.

The distribution of NV was confirmed with fundus examination and FA using Optos 200T (Dunfermline, UK) Heidelberg Retina Angiograph 2 (Heidelberg Engineering, Heidelberg, Germany). The number of NV areas identified using a VRI slab (from ILM to 2000 μm: The field of view of the fundus will be described later) for foveal central and disc with OCTA images using OCT-S1 (Canon, Tokyo, Japan) were compared with those confirmed exact match. OCT-S1 is an ultra-widefield OCT platform. OCT-S1 can capture OCTA over a wide range of 78° horizontally and 68° vertically (23 × 20 mm). NV was defined as blood flow signals detected within the vitreous cavity of 2000 μm from the ILM using OCT-S1. Three experienced retina physicians (Y.H., A.S., and M.T.) were blinded to the patient clinical status. The enface area images were compared using ImageJ (National Institutes of Health, Bethesda, MD; https://rsb.info.nih.gov/ij/index.html). Three experienced retina physicians (Y.H., A.S., and M.T.), blinded to the patient's clinical condition, used ImageJ (National Institutes of Health, Bethesda, MD) to encircle the NV on the enface images and measure its area in pixels (NV area) (Figure 1).

The primary outcome measure is as follows: Comparison of the preoperative NV area between the two groups. The secondary outcome measures are as follows: (1) comparison of the postoperative NV area between the two groups; (2) comparison of the preoperative NV area and the postoperative NV area in each group; and (3) correlation between the preoperative NV area and BCVA at latest visit.

### 2.2. Statistical Analysis

All statistical analyses were performed with EZR (Version 1.60, Saitama Medical Center, Jichi Medical University, Saitama, Japan), which is used for the calculation of R. More precisely, it is a modified version of R commander, which is designed to add statistical functions frequently used in biostatistics [21]. Student's paired *t*-test was used to test for significant differences in the mean values between before surgery and after surgery within the same group. The Mann–Whitney *U* test was used to test for significant differences in the mean values between the two groups, with the correlation between the measured values of the various quantities examined using Spearman's correlation coefficient. Values of *p* < 0.05 were considered statistically significant.

## 3. Results

### 3.1. Preoperative Characteristics

A total of 37 eyes of 31 patients underwent 3-port, 25 or 27-gauge pars plana diagnostic vitrectomy (PPV); there were 27 eyes of 23 male patients and 12 eyes of 8 female patients. The VH group consisted of 8 patients with 11 eyes (4 men and 4 women) with a mean age of 49.7 ± 14.2 years, while the control group consisted of 23 patients with 26 eyes (19 men and 4 women) with a mean age of 56.9 ± 11.8 years. Before surgery, BCVA was 0.89 ± 0.78 in the control group and 1.27 ± 0.64 in the VH group. TRD was observed in three eyes in the control group and six eyes in the VH group. 15 eyes in the control group and 8 eyes in the VH group received anti-VEGF intravitreal injections within 1 month of surgery. Before surgery, patients underwent 2.5 ± 1.9-quadrant laser retinal PC in the control group and 1.8 ± 1.5-quadrant laser retinal PC in the VH group (Table 1).

### 3.2. Intraoperative Detail

Nineteen eyes in the control group and 7 eyes in the VH group underwent concomitant cataract surgery (*p*=0.44). Vitrectomy was performed in all cases, and the proliferative membrane was removed using intraocular forceps. PC was performed from the posterior pole to the peripheral retina (excluding the arcade vessels) in areas without PC scars. The number of PC shots and the irradiation site were left to the discretion of the surgeon. The number of PC shots performed during surgery was 1190.2 ± 454.5 shots in the control group and 963.3 ± 404.7 shots in the VH group (*p*=0.19). Three eyes in the control group and six eyes in the VH group were treated for TRD. 10 eyes in the control group and 7 eyes in the VH group were injected with air or sulfur hexafluoride (SF6) gas (*p*=0.17). One eye in the VH group received intraocular injection of silicone oil (*p*=0.14) (Table 2).

### 3.3. OCTA Image Analysis

**Figure 2:**
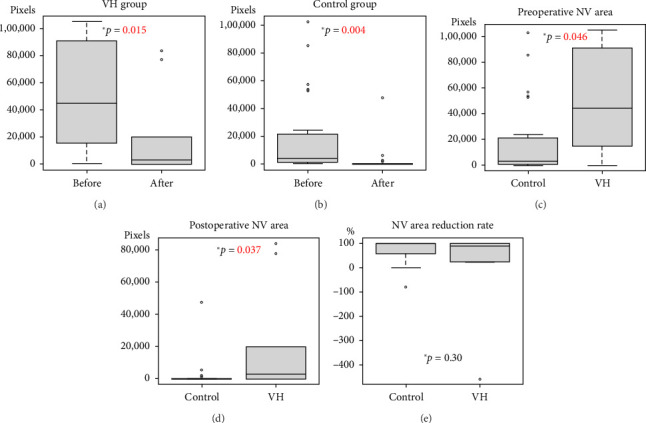
Comparison of retinal neovascular (NV) area (pixels). (a) Preoperative NV area vs postoperative NV area in the vitreous hemorrhage (VH) group. (b) Preoperative NV area vs postoperative NV area in the control group. (c) Control group vs VH group before surgery. (d) Control group vs VH group after surgery. (e) NV area reduction rate after operation compared to before surgery. The right is the control group, and the left is the VH group.

The preoperative retinal neovascular area was 50,233.7 ± 38,581.1 (pixels) in the VH group and 17,155.4 ± 27,950.2 (pixels) in the control group, showing a significant difference (*p*=0.046). The postoperative retinal neovascular area was 36,315.7 ± 44,311.8 (pixels) in the VH group and 2261.0 ± 9072.2 (pixels) in the control group, showing a significant difference (*p*=0.046), but there was no significant difference in the reduction rate of the neovascular area before and after surgery (*p*=0.30). In both the control group and the VH group, the postoperative retinal neovascular area was significantly reduced compared to the preoperative retinal neovascular area (Figure 2).

**Figure 3:**
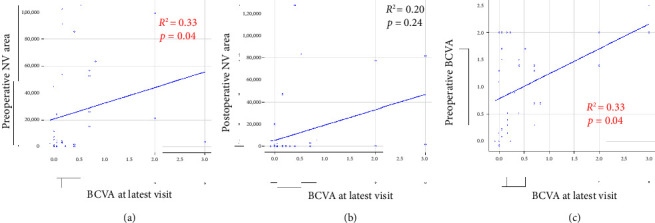
Spearman's rank correlation coefficient. (a) Correlation between the preoperative NV area and best-corrected visual acuity (BCVA) at latest visit. (b) Correlation between the postoperative NV area and BCVA at latest visit. (c) Correlation between BCVA before surgery and BCVA at latest visit.

At latest visit, BCVA was 0.36 ± 0.67 in the control group and 0.76 ± 0.89 in the VH group (*p*=0.12). The mean postoperative follow-up period was 15.2 ± 11.4 months in the control group and 21.2 ± 5.8 months in the VH group (*p*=0.087). In the control group, BCVA at latest visit was significantly improved compared with preoperative BCVA (*p*=0.0016). In the VH group, BCVA at latest visit did not show significant improvement compared with preoperative BCVA (*p*=0.084). In 37 eyes, including both the VH and control groups, BCVA at latest visit was significantly correlated with preoperative BCVA (Spearman's correlation coefficient: *R*^2^ = 0.33, *p*=0.04). In 37 eyes, including both the VH and control groups, BCVA at latest visit was significantly correlated with the preoperative NV area (Spearman's correlation coefficient: *R*^2^ = 0.33, *p*=0.04). In 37 eyes, including both the VH and control groups, BCVA at latest visit was not significantly correlated with the postoperative NV area (Spearman's correlation coefficient: *R*^2^ = 0.20, *p*=0.24) (Figure 3).

## 4. Discussion

In this retrospective case series, we used wide-angle OCTA by using the wide-angle OCTA enface images to quantify enface and predict VH after vitreous surgery for PDR. In PDR that develops VH after vitrectomy surgery, the appearance of NV seen on pre- and postoperative OCTA is significantly more extensive than in cases that do not develop postoperative VH, and OCTA may be useful for preoperative evaluation.

These findings suggest that quantitative assessment with enface OCTA may correlate with the activity of retinal NV in PDR, further expanding the potential of OCTA in the perioperative period. The benefits of this result are not only informed consent for patients but also that surgeons and medical teams can be aware of the risks of the surgery in advance, and ultimately, it creates a good opportunity to discuss the surgical method and procedure.

Previous reports described that enface images were taken with wide-angle OCTA, and there are few studies that focus on NV in the vitreous cavity [12, 14–16].

Jung et al. described a correlation between changes in the foveal avascular zone in DR patients using quantitative image evaluation with enface OCTA [22]. Furthermore, several investigations using artificial intelligence have also begun in the field of OCTA in intraocular disease, and several studies have been reported [23, 24].

Heinke et al. reported that, speaking only of high-precision OCTA, it is already superior in both sensitivity and specificity to humans in diagnosing age-related macula degeneration [25].

Our study this time was a very primitive manual method study by investigators, who used widefield OCTA image changes to blot the area of NV before and after surgery and compare. If the image accuracy will be further improved, it may become possible to use AI for predictive diagnosis of perioperative complications in DR in next generation investigation.

As for clinical implications, we were able to confirm that NV of PDR can be detected noninvasively using wide-angle OCTA, which can avoid invasive FA [26]. In addition, a team is also enabled to objectively assess the risk of PDR surgery.

Limitations include the possibility of bias in the manual measurements of the retinal neovascular area by investigators and the small number of cases.

This study has several limitations. First, because it is a retrospective study, the possibility of selection bias cannot be denied. In addition, because the analysis was performed for each eye, both eyes of the same patient were analyzed separately. Therefore, the sample size was small, and correlation between the two eyes may have influenced the results. Finally, there is a possibility that the researchers' manual measurement of the neovascular area may have been biased. We tried to reduce this bias by having three doctors measuring it.

## 5. Conclusion

In PDR developing VH after vitrectomy surgery, the appearance of NV seen on pre- and postoperative enface widefield OCTA is significantly more extensive than in cases that do not develop postoperative VH, and enface widefield OCTA may be useful for preoperative evaluation. If the image accuracy will be further improved, it may become possible to use AI for predictive diagnosis of perioperative complications in DR in next generation investigation.

## Figures and Tables

**Table 1:** Patient background.

	Control group	VH group	*p* value
Patient	23	8	
Eyes	26	11	
Age	56.9 ± 11.8	49.7 ± 14.2	0.16
Sex	Male: 19, female: 4	Male: 4, female: 4	**0.04**
BCVA before surgery (logMAR)	0.89 ± 0.78	1.27 ± 0.64	0.13
Number of eyes with TRD	3	6	**0.0064**
Use of anti-VEGF drugs (within 1 month before surgery)	15	8	0.39
How many quadrants were PC performed before surgery? (0∼4)	2.5 ± 1.9	1.8 ± 1.5	0.17

*Note:* Bold values indicate statistically significant differences between groups (*p* < 0.05).

**Table 2:** Inoperative detail.

	Control group	VH group	*p* value
Number of eyes who underwent cataract surgery	19	7	0.44
Number of laser shots performed during surgery (average)	1190.2 ± 454.5	963.3 ± 404.7	0.19
Number of eyes injected with air or SF6 gas	10	7	0.17
Number of eyes injected with silicone oil	0	1	0.14

## Data Availability

The data that support the findings of this study are available from the corresponding author upon reasonable request.
